# Supplementary material for: Vibrio cholerae motility is associated with inter-animal transmission
Source: Nat Commun. 2025 Aug 27;16:7989. doi: 10.1038/s41467-025-62984-4 (PMC12391452; doi:10.1038/s41467-025-62984-4)
Supplement: Supplementary file 1 — Supplementary Information [file 41467_2025_62984_MOESM1_ESM.pdf]

Supplemental figures

Vibrio cholerae motility is associated with inter-animal transmission

Ian W. Campbell*[1], Ruchika Dehinwal*[1], Alexander A. Morano[1], Katherine G. Dailey[1], Franz G. Zingl[1], Matthew K. Waldor[1,2]#

[1] Division of Infectious Diseases at Brigham & Women's Hospital, and Department of Microbiology at Harvard Medical School, Boston, MA
[2] Howard Hughes Medical Institute, Boston, MA, USA

*Ian W. Campbell and Ruchika Dehinwal contributed equally to this work
#Correspondence: mwaldor@bwh.harvard.edu

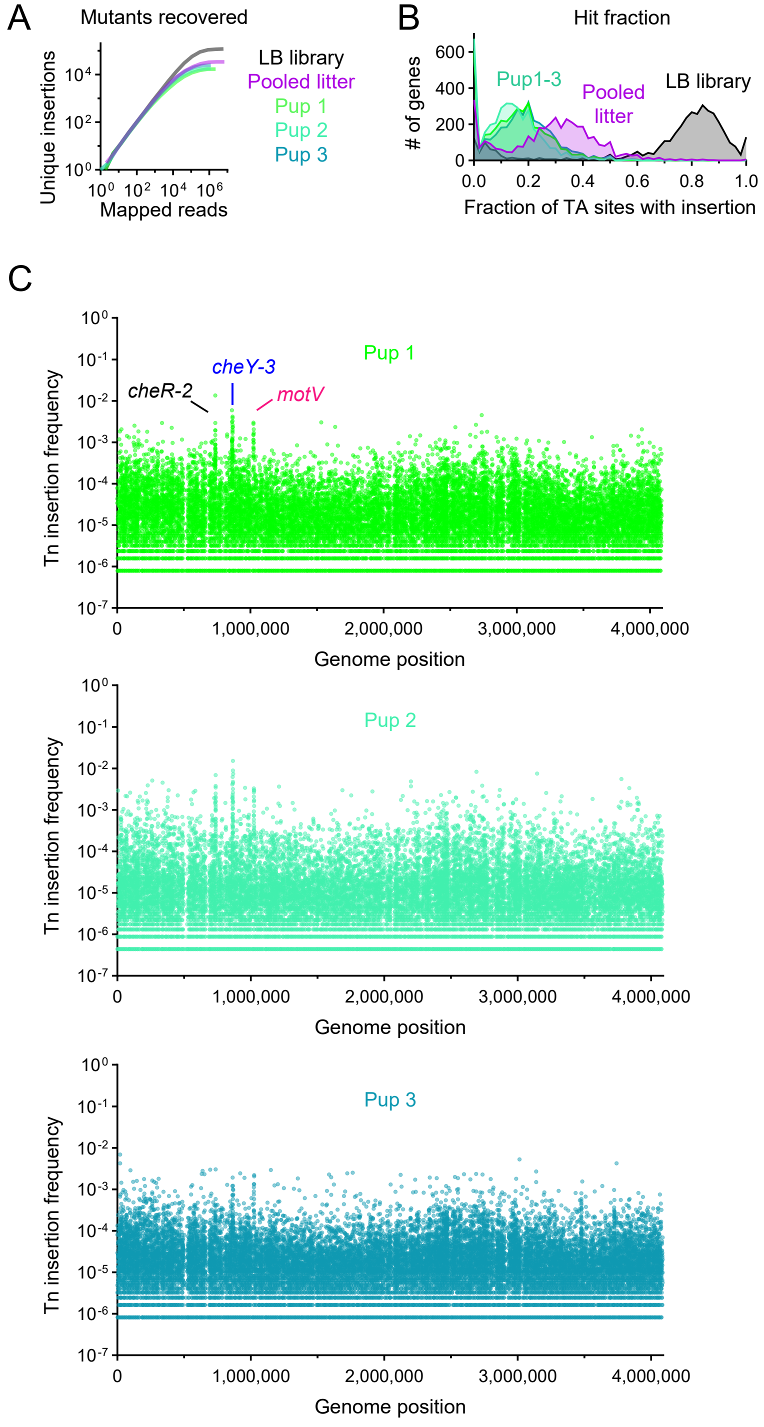

Supplemental figure 1 | Pooling a litter of CD1 pups increases the fraction of the transposon library recovered after infection. Extended data from Fig 1. P4 CD1 mice were intragastrically inoculated with ~5x10^{7} CFU of a mariner transposon library. 18-hours later, *V. cholerae* from the SIs of individual pups or the pooled SIs of an entire litter (10 pups) were outgrown overnight on LB, and then sequenced. For comparison, the same library was passaged overnight on LB media (LB library). **A,** The reads from each library were randomly sampled, and the number of unique insertion sites were plotted to depict diversity across sampling depths. **B,** Histogram of fraction of possible insertion sites in each gene disrupted by

a transposon insertion. TA sites are sites with a TA dinucleotide. Mariner transposons primarily integrate at TA sites, and data throughout the manuscript is filtered only to include transposons integrated at TA sites. **C,** Transposon insertion frequency across the genome, highlighting increased insertion frequency in *cheR-2, cheY-3,* and *motV* from individual CD1 pups. Source data are provided as a Source Data file.

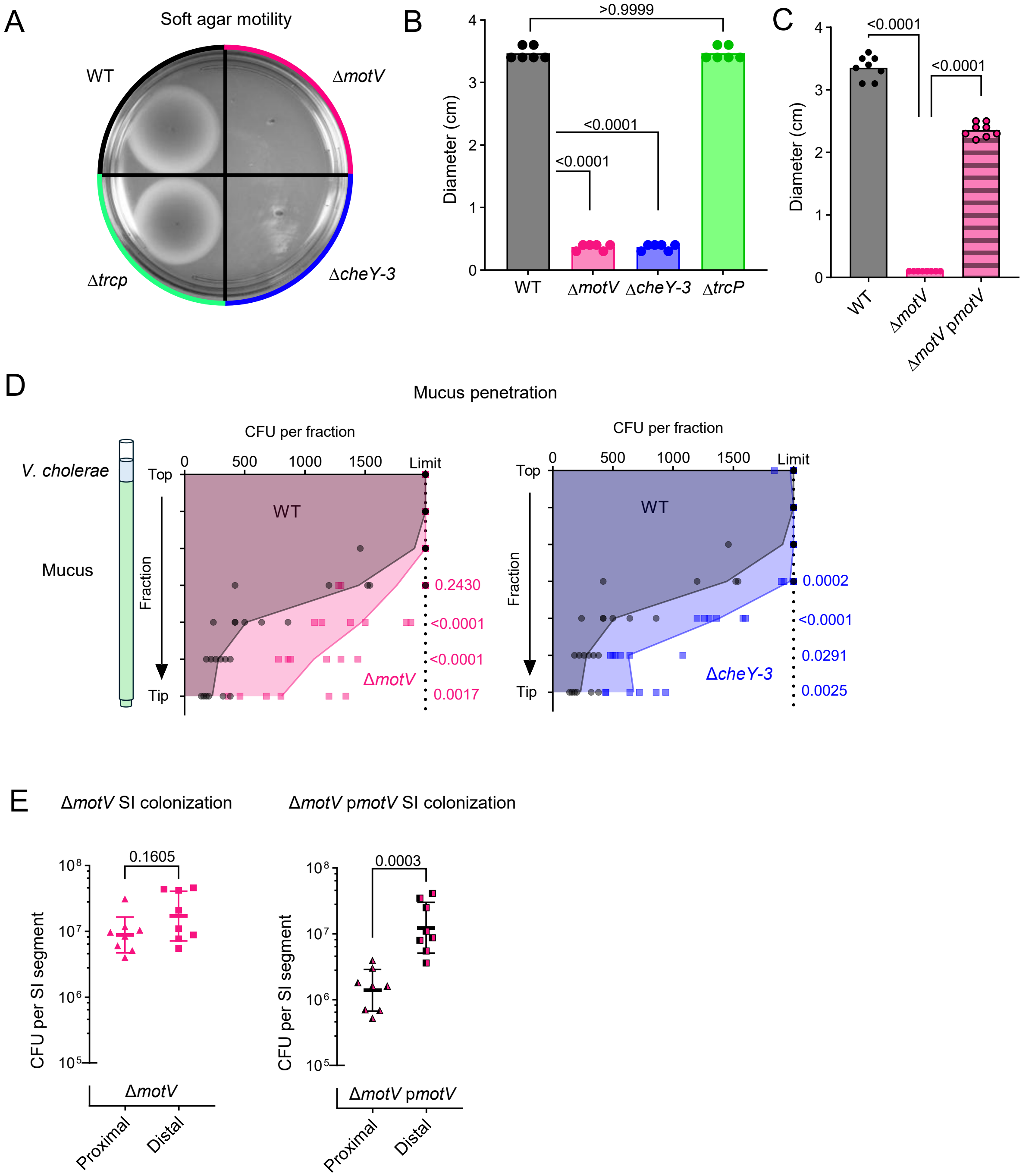

Supplemental figure 2 | Extended observations and complementation of the Δ*motV* mutant. Extended data related to Fig 2. **A-C,** Motility of indicated *V. cholerae* strains in soft agar plates. **A,** Image of a representative plate. **B-C,** Diameter of motility zones. A Δ*trcp* mutant is included as an additional control. Complementation of the Δ*motV* mutant with a plasmid-borne copy of *motV* (p*motV*) restores motility in soft agar. Mean and individual replicates. *n* = 6-8 colonies per strain. One-way ANOVA with Dunnett's multiple comparison correction. **D,** Motility of indicated *V. cholerae* strains in a mucus column. Strains were layered on top of a 1% mucus column and incubated for 30 minutes at 37 °C. 100 µl fractions were collected and plated to determine penetration towards the tip of the column. Upper limit to CFU detection was 2,000 cells. Mean and standard deviation. *n* = 6 columns per strain.

Two-way ANOVA compared to WT. **E,** CD1 mice were intragastrically inoculated with the Δ*motV* mutant or Δ*motV* complemented with p*motV*, and 18-hours later CFU was enumerated from the indicated SI segments. Geometric mean and standard deviation. Two-way Mann-Whitney test. *n* = 8 pups (1 litter) per strain. Source data are provided as a Source Data file.

A

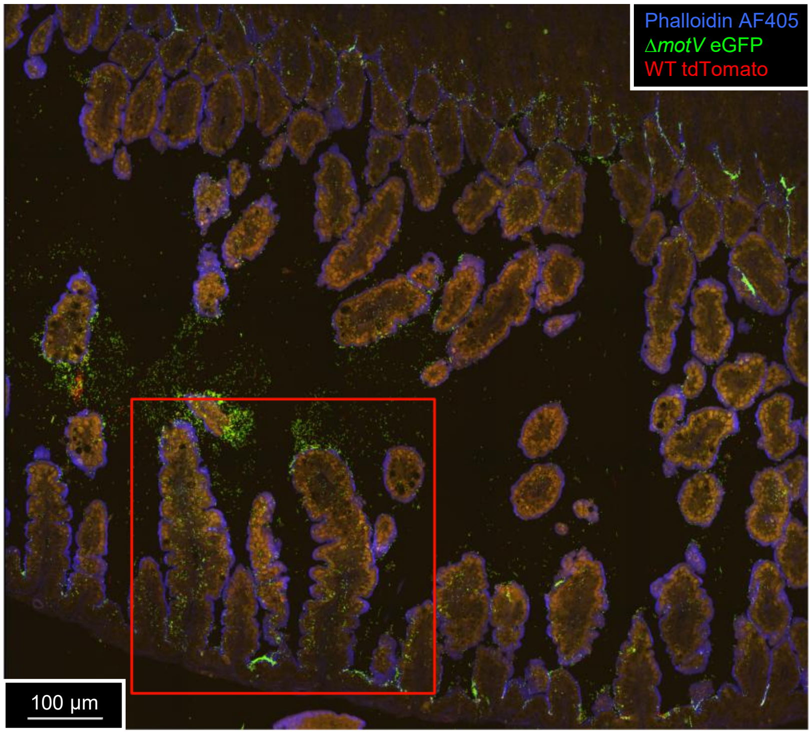

B

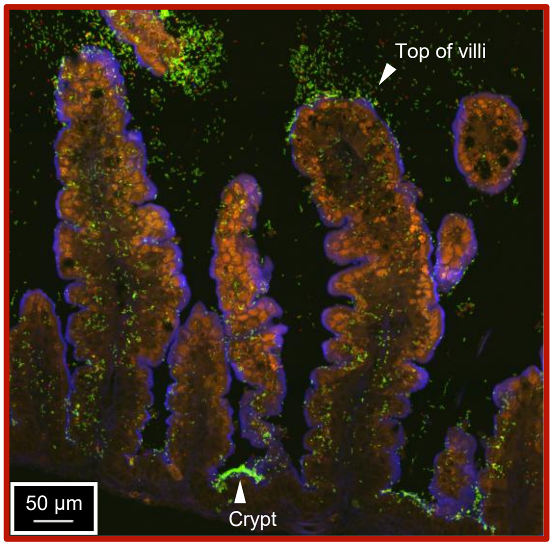

Supplemental figure 3 | Large image of *V. cholerae* localization in the SI. Related to Fig 2. **A,** Large image of a 10 μm cryosection of the medial SI 18-hours after inoculation with ~2 x 10^6 CFU of a 1:1 ratio of WT *lacZ::tdTomato V. cholerae* (red) and Δ*motV lacZ::eGFP V. cholerae* (green). Shown are the villi of the SI on either side of the lumen. Sections were stained with AF405-conjugated phalloidin (blue) and imaged on a spinning disk confocal microscope. **B,** Zoomed-in portion of **A** (400 x 400 μm) showing the top of villi and crypts of the SI in more detail; outlined in red on **A**.

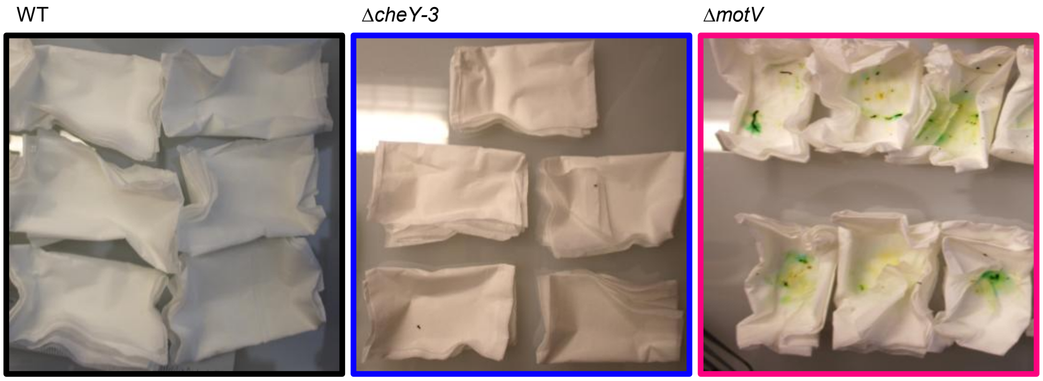

Supplemental figure 4 | Deletion of *motV* increased infant mouse diarrheal discharge during infection with *V. cholerae*. CD1 pups were intragastrically inoculated with the indicated *V. cholerae* strains and individually housed for 18-hours in the pictured bedding. Each tissue is the bedding of a single infected animal. The *V. cholerae* inoculum is dyed green to assist with intragastric gavage, and subsequent discharge of the dye stains the bedding, assisting in visualizing diarrhea discharge.

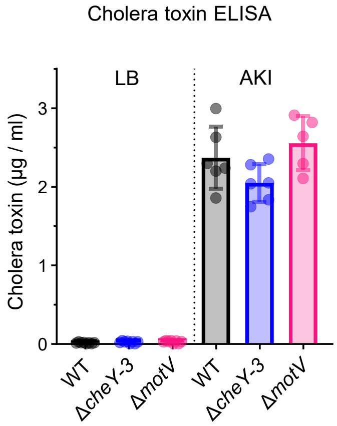

Supplemental figure 5 | *motV* deletion does not change cholera toxin production in culture. ELISA measurement of cholera toxin following culture. Mean and standard deviation. Not significant by one-way ANOVA with Tukey's multiple comparison correction. *n* = 5-8 cultures per strain per growth condition. Source data are provided as a Source Data file.

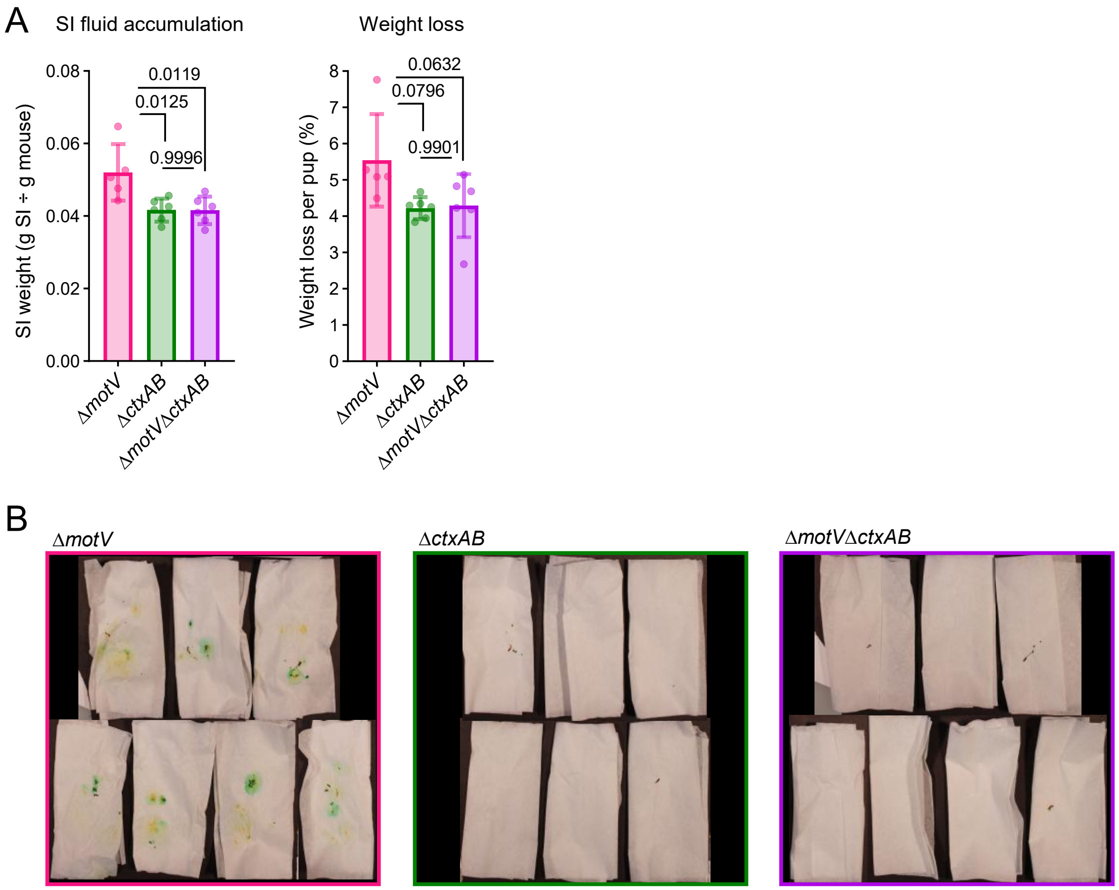

Supplemental figure 6 | Deletion of *ctxAB* decreases the diarrheaogenicity of the Δ*motV* mutant. CD1 pups were intragastrically inoculated with the indicated *V. cholerae* strains and individually housed for 18-hours. **A,** Fluid accumulation in the SI was measured by comparing the weight of the animal to the weight of the SI. Weight loss was determined by comparing animal weight before and after infection. Mean and standard deviation. One-way ANOVA with Tukey's multiple comparison correction. *n* = 17 pups (2 litters) randomized between strains. **B,** Diarrheal discharge was visualized as staining of the bedding that housed the infant mice during infection. Each tissue is the bedding of a single infected animal. The *V. cholerae* inoculum is dyed green to assist with intragastric gavage, and subsequent discharge of the dye stains the bedding. *n* = 20 pups (2 litters) randomized between strains. Source data are provided as a Source Data file.
