# Supplementary material for: Vibrio cholerae motility is associated with inter-animal transmission
Source: Nat Commun. 2025 Aug 27;16:7989. doi: 10.1038/s41467-025-62984-4 (PMC12391452; doi:10.1038/s41467-025-62984-4)
Supplement: Supplementary file 2 — Description of Additional Supplementary Files [file 41467_2025_62984_MOESM2_ESM.pdf]

Supplementary Data Legends

Supplementary Data 1| Tn-seq analysis of *V. cholerae* infant mouse infection. Related to Fig. 1. Results of Tn-seq analysis. 10 P4 CD1 mice were intragastrically inoculated with ~5x10^7 CFU of a mariner transposon library. 18-hours later, *V. cholerae* from the pooled SIs of an entire litter (10 pups) were outgrown overnight on LB, and then sequenced. For comparison, the same library was passaged overnight on LB media (LB library). For every gene, fold-change in gene insertion frequency (“Log2FoldChange”) was compared between the pooled litter and LB library with *P* value derived from a two-sided Mann-Whitney test (“MWU_P-value”).

Supplementary Data 2| Bacterial strains used in this study. A list of the strains used in this study, including the source of the strain.

Supplementary Data 3| Primers used in this study. A list of the primers used in this study, including the sequence of the primer and notes regarding the application of the primer.
