# Supplementary material for: Vibrio cholerae motility is associated with inter-animal transmission
Source: Nat Commun. 2025 Aug 27;16:7989. doi: 10.1038/s41467-025-62984-4 (PMC12391452; doi:10.1038/s41467-025-62984-4)
Supplement: Supplementary file 4 — Reporting Summary [file 41467_2025_62984_MOESM4_ESM.pdf]

nature portfolio

Corresponding author(s): Matthew K. Waldor

Last updated by author(s): Jul 23, 2025

Reporting Summary

Nature Portfolio wishes to improve the reproducibility of the work that we publish. This form provides structure for consistency and transparency in reporting. For further information on Nature Portfolio policies, see our Editorial Policies and the Editorial Policy Checklist.

Statistics

For all statistical analyses, confirm that the following items are present in the figure legend, table legend, main text, or Methods section.

n/a	Confirmed	
☐	☒	The exact sample size (*n*) for each experimental group/condition, given as a discrete number and unit of measurement
☐	☒	A statement on whether measurements were taken from distinct samples or whether the same sample was measured repeatedly
☐	☒	The statistical test(s) used AND whether they are one- or two-sided<br>*Only common tests should be described solely by name; describe more complex techniques in the Methods section.*
☒	☐	A description of all covariates tested
☐	☒	A description of any assumptions or corrections, such as tests of normality and adjustment for multiple comparisons
☐	☒	A full description of the statistical parameters including central tendency (e.g. means) or other basic estimates (e.g. regression coefficient) AND variation (e.g. standard deviation) or associated estimates of uncertainty (e.g. confidence intervals)
☐	☒	For null hypothesis testing, the test statistic (e.g. *F*, *t*, *r*) with confidence intervals, effect sizes, degrees of freedom and *P* value noted<br>*Give P values as exact values whenever suitable.*
☒	☐	For Bayesian analysis, information on the choice of priors and Markov chain Monte Carlo settings
☒	☐	For hierarchical and complex designs, identification of the appropriate level for tests and full reporting of outcomes
☒	☐	Estimates of effect sizes (e.g. Cohen's *d*, Pearson's *r*), indicating how they were calculated

Our web collection on statistics for biologists contains articles on many of the points above.

Software and code

Policy information about availability of computer code

Data collection	Tn-seq data were collected using an Illumina NextSeq.
Data analysis	Data analysis was performed using CLC Genomics Workbench version 12.0.2 (Trim reads 2.3 and Read mapping 1.6 functions), R version 4.4.2 (RTISAn Tn-seq analysis), GraphPad Prism, and Excel. R scripts were previoulsy described in peer reviewed literature, which is cited.

For manuscripts utilizing custom algorithms or software that are central to the research but not yet described in published literature, software must be made available to editors and reviewers. We strongly encourage code deposition in a community repository (e.g. GitHub). See the Nature Portfolio guidelines for submitting code & software for further information.

Data

Policy information about availability of data

All manuscripts must include a data availability statement. This statement should provide the following information, where applicable:

- Accession codes, unique identifiers, or web links for publicly available datasets
- A description of any restrictions on data availability
- For clinical datasets or third party data, please ensure that the statement adheres to our policy

Sequencing reads from Tn-seq are deposited in the Sequencing Read Archive (SRA) under accession no. PRJNA1247499 [https://www.ncbi.nlm.nih.gov/bioproject/PRJNA1247499] . Source data are provided with this paper as a source data file.

Research involving human participants, their data, or biological material

Policy information about studies with human participants or human data. See also policy information about sex, gender (identity/presentation), and sexual orientation and race, ethnicity and racism.

Reporting on sex and gender	N/A
Reporting on race, ethnicity, or other socially relevant groupings	N/A
Population characteristics	N/A
Recruitment	N/A
Ethics oversight	N/A

Note that full information on the approval of the study protocol must also be provided in the manuscript.

Field-specific reporting

Please select the one below that is the best fit for your research. If you are not sure, read the appropriate sections before making your selection.

- [x] Life sciences
- [ ] Behavioural & social sciences
- [ ] Ecological, evolutionary & environmental sciences

For a reference copy of the document with all sections, see nature.com/documents/nr-reporting-summary-flat.pdf

Life sciences study design

All studies must disclose on these points even when the disclosure is negative.

Sample size	Sample sizes were determined by the variation observed in previous studies and preliminary experiments. For Tn-seq, initially 3 animals were sequenced separately. Based on the noise observed in this preliminary experiment (Fig S1), the subsequent experiment was increased to pool an entire litter of 10 pups. Based on the magnitude of the fold-change in mutant fitness observed in the Tn-seq data it was determined that a single litter of ~10 pups was sufficient for subsequent colonization experiments using the motV mutant strain. Transmission experiments were repeated with 3 litters of ~10 pups per bacterial strain; as the transmission experiment is a new technique we could not know the noise expected between experimental groups.
Data exclusions	No data were excluded from this study.
Replication	All attempts at replication were successful. Replicates are described in figure legends.
Randomization	Pups were randomly assigned to experimental groups.
Blinding	Blinding was not used in these studies because the quantifiable variables (CFU, sequencing reads, weight loss, diarrheal discharge) are not subject to investigator bias.

Reporting for specific materials, systems and methods

We require information from authors about some types of materials, experimental systems and methods used in many studies. Here, indicate whether each material, system or method listed is relevant to your study. If you are not sure if a list item applies to your research, read the appropriate section before selecting a response.

Materials & experimental systems

n/a	Involved in the study	
[ ]	[x]	Antibodies
[x]	[ ]	Eukaryotic cell lines
[x]	[ ]	Palaeontology and archaeology
[ ]	[x]	Animals and other organisms
[x]	[ ]	Clinical data
[x]	[ ]	Dual use research of concern
[x]	[ ]	Plants

Methods

n/a	Involved in the study	
[x]	[ ]	ChIP-seq
[x]	[ ]	Flow cytometry
[x]	[ ]	MRI-based neuroimaging

Antibodies

Antibodies used	Rabbit anti-CT polyclonal antibody (Abcam; catalog no. ab123129)
Validation	Previously described in Fakoya et al. 2022 (https://doi.org/10.1128/mbio.00539-22)

Animals and other research organisms

Policy information about studies involving animals; ARRIVE guidelines recommended for reporting animal research, and Sex and Gender in Research

Laboratory animals	CD1 (Crl:CD1(ICR)) dams with litters (mixed sex) were purchased from Charles River Laboratories (strain #022) and pups were infected at postnatal day 3-5. Mice were housed in a biosafety level 2 (BSL2) facility under specific pathogen free conditions at 68-75 °F, with 30-50% humidity, and a 12 h light/dark cycle.
Wild animals	No wild animals were used in this study.
Reporting on sex	Mixed sex pups were used. Sex was not determined. Sex is not an observable trait in the infant mice used in this study.
Field-collected samples	No field-collected samples were used in this study.
Ethics oversight	Animal studies were conducted at the Brigham and Women's Hospital in compliance with the Guide for the Care and Use of Laboratory Animals and according to protocols reviewed and approved by the Brigham and Women's Hospital Institutional Animal Care and Use Committee (protocol 2016N000416).

Note that full information on the approval of the study protocol must also be provided in the manuscript.

Plants

Seed stocks	N/A
Novel plant genotypes	N/A
Authentication	N/A
